# Mobile phone-based systems for community-led injury response and coordination: a scoping review protocol

**DOI:** 10.1097/SP9.0000000000000040

**Published:** 2025-05-13

**Authors:** Brandon George Smith, Thomas Edmiston, Laura Hobbs, Michael Bath, Katharina Kohler, Saleyha Ahsan, Isla Kuhn, Tonny Luggya, Shobhana Nagraj, Sara Venturini, Cornelius Sendagire, Daphne Kabatoro, Almas Khattak, Charlotte Jane Whiffin, Peter John Hutchinson, Tom Bashford, Tariq Khan, Arthur Kwizera

**Affiliations:** a International Health Systems Group, Department of Engineering, University of Cambridge, Cambridge, UK; b Cambridge Public Health Interdisciplinary Research Centre, University of Cambridge, Cambridge, UK; c NIHR Global Health Research Group on Acquired Brain and Spine Injury, University of Cambridge, Cambridge, UK; d Cambridge University Hospitals NHS Foundation Trust, Addenbrooke’s Hospital, Cambridge, UK; e Department of Anaesthesia, East and North Hertfordshire NHS Trust, Stevenage, UK; f North Middlesex University Hospital NHS Foundation Trust, London, UK; g Royal College of Surgeons of England, London, UK; h Department of Anaesthesia, Cambridge University Hospitals NHS Foundation Trust, Cambridge, UK; i Cambridge University Medical Library, School of Clinical Medicine, University of Cambridge, Cambridge, UK; j Department of Anaesthesia and Critical Care, Makerere University College of Health Sciences, Kampala, Uganda; k Department of Public Health & Primary Care, University of Cambridge, Cambridge, UK; l East London NHS Foundation Trust, London, UK; m Division of Neurosurgery, Department of Clinical Neurosciences, Addenbrooke’s Hospital and University of Cambridge, Cambridge, UK; n Community Medicine and Research, Northwest School of Medicine, Peshawar, Pakistan; o College of Health, Psychology and Social Care, University of Derby, Derby, UK; p Neurosurgery, Northwest General Hospital and Research Center, Peshawar, Pakistan

**Keywords:** community responders, injury, mobile phones, trauma

## Abstract

Traumatic injuries remain a leading cause of preventable death globally, and continue to burden global healthcare services, particularly in low-resource settings. Mobile phone-based community injury response and coordination (mCIRC) systems represent a promising solution in facilitating rapid identification of injured persons, and coordinating a community-led response as an alternative or adjunct to a formal emergency service. mCIRC systems may use technologies such as geolocation and push notifications to mobilize trained responders in the vicinity of the incident, ensuring timely intervention before professional medical services arrive. This scoping review aims to provide a comprehensive overview of the existing evidence on the effectiveness and implementation of mCIRC systems in response to trauma as well as other medical emergencies, such as out-of-hospital cardiac arrest. We will evaluate their deployment in both high- and low-resource settings. In particular, the review will assess how these systems improve health outcomes of patients, such as reducing mortality and morbidity, and the feasibility and uptake of such systems by the global community. Additionally, the review will explore the operational challenges and facilitators of implementing such systems, particularly in low- and middle-income countries where healthcare infrastructure is often limited. This review will offer a comprehensive insight into the role of mobile technologies in improving trauma care at the community level, highlighting possible avenues for future research in this domain.

## Introduction

The management of medical emergencies, particularly those arising from road traffic accidents, falls, burns, and drowning, remains a significant public health challenge, contributing to high rates of preventable death and long-term disability globally. The effective management of these emergencies is especially difficult in low-resource settings, where limited healthcare infrastructure and delayed access to emergency services often exacerbates the consequences of such injuries.

While a centralized and coordinated emergency service model adopted in many settings offers one solution to address this challenge, these services can become overwhelmed when faced with a surge in cases (e.g. following a mass casualty) or a disruption to infrastructure (e.g. following a natural hazard), leading to delays in emergency response and timely care for the injured. Furthermore, it is noteworthy that prehospital care, including structured emergency medical services with adequate coverage, is minimal or absent in many low-resource settings, leaving a substantial gap in critical early medical intervention capabilities^[1,2]^. Previous evidence has highlighted the critical role of community and bystander involvement in prehospital care, which can significantly reduce mortality rates and improve patient outcomes^[3]^. Indeed, early intervention by community members or trained bystanders at the scene of an injury is crucial in stabilizing patients, providing life-saving first aid, and facilitating faster transportation to medical facilities^[4]^.

**Highlights**

- Mobile phone-based community response systems have been shown to enable rapid identification and mobilization of responders for out-of-hospital cardiac arrests, providing timely interventions for such emergencies.
- This scoping review will map evidence on the effectiveness of mobile response systems in facilitating and coordinating community-led responses to injury or trauma, identifying key facilitators and challenges for global implementation.
- Insights from this review will guide future research on mobile phone-based community injury response and coordination (mCIRC) systems, with a focus on low-resource settings.

Mobile phone-based community injury response and coordination (mCIRC) systems, such as GoodSAM^[5,6]^ and PulsePoint^[7]^, have emerged as a promising solution to strengthening the prehospital response to out-of-hospital cardiac arrests (OOHCA) in high-income settings^[5–7]^. These systems leverage pervasive cellular mobile networks, geolocation services, smartphone applications, and real-time notification functionality to facilitate the identification of injured persons and the alerting of trained responders in the vicinity. Yet despite the potential benefits of mCIRC systems, there is limited comprehensive evidence on their effectiveness and implementation, particularly in resource-limited settings.

This scoping review aims to synthesize the existing research on mCIRC systems, evaluate their impact on health outcomes, and explore the operational challenges and facilitators to their implementation, particularly in low- and middle-income countries (LMICs). In particular, we will evaluate the viability of these interventions for improving care for trauma patients.

## Methods

This scoping review aims to synthesize the existing evidence on the use of mobile phone-based community-led response and coordination systems for trauma globally, to assess their impact on response times, morbidity, and mortality, and to explore barriers (e.g. infrastructural or financial costs, technology requirements, behavioral and cultural challenges) and facilitators (e.g. charitable or state funding, provision of technology, remuneration of first responders) to their implementation.

This scoping review seeks to address the following questions:

- What are the key characteristics of community-led interventions enabled by mobile phone technologies in injury response and coordination?
- What is the effectiveness of mobile phone-based injury response and coordination systems in improving health outcomes (e.g. reducing mortality and morbidity) for trauma victims?
- Where reported, how do these systems impact response times in different settings, including urban, rural, and remote areas?
- What barriers and facilitators exist for the implementation of mobile phone-based emergency response systems in various contexts?

The reporting of this scoping review will be done in accordance with the Preferred Reporting Items for Scoping Reviews and Meta-Analyses extension for scoping reviews^[8]^, and follows a systematic process of: Study Identification, Screening, Eligibility, Inclusion, and Analysis. Review objectives, eligibility criteria, and preliminary study characteristics to be extracted were determined *a priori*.

### Inclusion criteria

Any published original research with full texts available in English, including: primary studies (all study designs), protocols, reports, editorials, opinion articles, letters, conference abstracts, theses, and book chapters are eligible for inclusion. Articles will be excluded if they are (a) secondary research (i.e. reviews); (b) are not reported in English; or (c) describe efforts conducted in a non-healthcare context or controlled environment. Grey literature sources including doctoral dissertations, conference proceedings, commercial reports and press releases, government reports, and preprints will be searched via sources such as ProQuest, OpenGrey, Google Scholar, and relevant institutional repositories to capture unpublished research pertinent to this review. Published material from grey literature sources will be appraised to determine quality of the information reported using the six criteria of the AACODS checklist (Authority, Accuracy, Coverage, Objectivity, Date, Significance)^[9]^.

The population/participants, concept, and context framework^[10,11]^ outlines the planned search strategy.

### Participants/population

The population of interest for this review includes both the injured individuals requiring emergency medical intervention and the trained responders (both laypersons and healthcare professionals) mobilized by mCIRC systems. Injured persons may suffer from various types of trauma, such as road traffic accidents, falls, burns, and drowning. The review will also encompass responders in diverse roles, from trained community members to formal emergency personnel, who provide timely intervention at the scene of the injury. The population scope is broad and not limited by specific demographic criteria such as age, gender, or socioeconomic status, as the focus is on individuals involved in emergency incidents where mobile phone-based systems have been deployed.

### Concept

The primary concept of this review is the use of mCIRC systems. These systems may leverage ubiquitous mobile phone technologies, such as SMS/text messaging, telephone or interactive voice response, unstructured supplementary service data or “Feature/Quick Codes,” and web-based or smartphone applications, to facilitate the rapid identification of injured persons and the coordination of a community-led response. The review will evaluate how these systems function to improve health outcomes, particularly through the mobilization of trained responders in the vicinity of the incident, to deliver care prior to the arrival of formal emergency services. The concept also includes an exploration of how these systems improve morbidity and mortality rates, reduce response times, and address operational challenges and facilitators in different settings.

### Context

The context for this review spans both high- and low-resource settings where mobile phone-based emergency systems have been implemented. The focus is on environments where these systems are deployed as an adjunct to, or substitute for, traditional emergency services. Particular emphasis will be placed on trauma, and setting of LMICs, where healthcare infrastructure is often limited and community-led initiatives are vital for timely emergency care. Studies will be included from urban, rural, and remote settings, capturing a wide range of socio-economic and infrastructural contexts to assess the scalability and adaptability of mCIRC systems in diverse environments.

### Search strategy, data charting, and synthesis of results

The search strategy (Table 1), co-developed with an academic clinical librarian (IK), will be applied to several key databases: *Ovid MEDLINE, Ovid Embase, Global Index Medicus, EBSCO Global Health, Scopus*, and *IEEE Xplore*. All articles from database inception will be retrieved. An example search strategy for Ovid MEDLINE, inclusive of MeSH (Medical Subject Headings) terms, is outlined in Table 1. A precursor proforma to guide study extraction and charting, and to be iteratively updated and refined as data charting progresses, is portrayed in Table 2.

**Table 1:** Example Ovid MEDLINE search strategy

1	(mobile phone* or smart phone* or cell* phone* or handy or iphone* or sms or text messag* or texting or “short messag* service*”).ti,ab,kw,kf.
2	exp Smartphone/
3	exp Cell Phone/
4	exp Text Messaging/
5	(mobile network* or mobile technology or apps or (phone adj3 application*)).ti,ab,kw,kf.
6	exp Mobile Applications/
7	(whatsapp or telegram* or “instant message” or signal).ti,ab,kw,kf.
8	mobile dispatch infrastructure.ti,ab,kw,kf. or exp Emergency Medical Dispatch/
9	mobile emergency medical dispatch tool.ti,ab,kw,kf.
10	or/1-9
11	(first responder* or first aid*).ti,ab,kw,kf.
12	exp Emergency Responders/
13	exp First Aid/
14	(layperson or lay person or lay people or passer-by* or bystander* or by-stander* or volunteer*).ti,ab,kw,kf.
15	(community responder* or community respons* or community injury respon*).ti,ab,kw,kf.
16	prehospital respon*.ti,ab,kw,kf.
17	or/12-16
18	(trauma* or accident* or fracture* or injuries or injured or cardiac arrest).ti,ab,kw,kf.
19	exp “Wounds and Injuries”/
20	Exp “Heart Arrest”/
21	rta or road traffic accident* or road traffic crash* or mva or motor vehicle accident*).ti,ab,kw,kf.
22	exp Accidents, Traffic/
23	or/19-22
24	10 and 17 and 23

**Table 2:** Precursor charting proforma indicating preliminary variables to be extracted and charted from retrieved articles

Context and settings
Urban/rural Country of implementation World Bank Country and Lending Group Classification (low-income; lower-middle-income; upper-middle-income; high-income)
Study design and characteristics
Study type Sample size Implementation duration Ethical approvals and governance
mCIRC system characteristics
Mobile technologies utilized (i.e. feature phone, smartphone) Software utilized (i.e. text messaging, voice calls, smartphone applications) Device ownership (i.e. personal device, provided device) Operational features (i.e. geolocation, push notifications) Network dependencies
Reported outcomes
mCIRC utilization (i.e. number of “calls” and “responses”) Number of community responders trained, if appropriate Response times Mortality and morbidity
Prospective barriers and facilitators to implementation
Operational/infrastructure/technical (i.e. network coverage, device accessibility, responder coverage, and coordination) Social/behavioral/cultural/contextual (i.e. community engagement, cultural acceptance of bystander aid, issues of equity and marginalization) Financial/economic (i.e. deployment and maintenance costs, funding) Policy/regulatory (i.e. liability policies and good samaritan laws, consent processes, data privacy)

Following data extraction and charting, findings will be analyzed to identify patterns and variations. Qualitative and quantitative evidence will be integrated in a narrative synthesis using a convergent approach; where reported, quantitative data will be summarized using descriptive statistics, and qualitative data will be thematically analyzed. This approach will enable a prospectively diverse evidence base to be synthesized, highlighting recurring themes and contextualization findings across varied settings. If sufficient homogenous quantitative data are available, supplementary statistical analyses may be explored.

## Discussion

The World Health Organization has advocated for the development of climate-resilient health systems and improved trauma care in humanitarian settings, with a particular focus on strengthening the prehospital response to injury at the community level^[12]^. The existing evidence base suggests that empowering local communities and bystanders to take an active role in the response to trauma, particularly in settings where emergency services are limited, is essential for improving health outcomes^[3,13–15]^. However, there remains limited evidence on the effectiveness and implementation of mCIRC systems, particularly in resource-limited settings.

This scoping review aims to assess all the current level of evidence in this area. By enabling timely intervention from nearby community members, such technologies offer a practical way to bridge the gap between injury occurrence and professional medical care, potentially reducing mortality and morbidity in both high- and low-resource settings. Given the relatively new and evolving nature of mobile-based community injury response and coordination, especially in low-resource settings, the number of available studies for inclusion may be limited. Additionally, commercially-driven developments of mCIRC systems, often reported through websites or multimedia platforms outside of traditional or grey literature repositories, may not be adequately captured by our scoping review methods. Thus, our ability to draw succinct, grounded conclusions (e.g. beneficial future avenues of research) may be limited. However, provisional screening of the literature suggests sufficient numbers will be present, and that an overview of the existing evidence base can be ascertained, and insights pertaining to the facilitators and barriers of mCIRC development and implementation, amongst their effectiveness, can be drawn.

Nonetheless, the purpose of the review is to identify the use cases of mCIRC systems implemented globally and reported in the literature base to date, offering a comprehensive overview of previous and ongoing initiatives conducted to date to aid the future development of an accessible, resilient, decentralized and device-stratified mCIRC system readily deployable in both high- and low-resource settings. Furthermore, it will be of interest to address the potential ethical challenges at play in the design and implementation of mCIRC systems, such as data privacy of both the enrolled first responder and injured persons, consent processes of all parties, and responder liability and safety with respect to their inherent function in directly introducing lay community responders to possibly hazardous live trauma scenes. By collating the evidence on these systems, the review will offer insights into the role of mobile technologies in improving trauma care at the community level and identify potential avenues for future research and development in this domain.

## Data Availability

Not applicable.
